# Multiplexed CRISPR technologies for gene editing and transcriptional regulation

**DOI:** 10.1038/s41467-020-15053-x

**Published:** 2020-03-09

**Authors:** Nicholas S. McCarty, Alicia E. Graham, Lucie Studená, Rodrigo Ledesma-Amaro

**Affiliations:** 1 Division of Biology and Biological Engineering, California Institute of Technology, Pasadena, CA 91125 USA; 2 Department of Bioengineering and Imperial College Centre for Synthetic Biology, Imperial College London, London, UK

**Keywords:** Genetic engineering, Gene expression, Synthetic biology

## Abstract

Multiplexed CRISPR technologies, in which numerous gRNAs or Cas enzymes are expressed at once, have facilitated powerful biological engineering applications, vastly enhancing the scope and efficiencies of genetic editing and transcriptional regulation. In this review, we discuss multiplexed CRISPR technologies and describe methods for the assembly, expression and processing of synthetic guide RNA arrays in vivo. Applications that benefit from multiplexed CRISPR technologies, including cellular recorders, genetic circuits, biosensors, combinatorial genetic perturbations, large-scale genome engineering and the rewiring of metabolic pathways, are highlighted. We also offer a glimpse of emerging challenges and emphasize experimental considerations for future studies.

## Abstract (web summary)

Multiplexed CRISPR technologies have recently emerged as powerful approaches for genetic editing and transcriptional regulation. Here the authors review this emerging technology and discuss challenges and considerations for future studies.

## Introduction

Most archaea and about half of bacteria carry the genes necessary for CRISPR–Cas adaptive immunity, which provides a memory of prior infections by encoding short DNA sequences into CRISPR (clustered regularly interspaced short palindromic repeats) loci within their genome. These prior infections are stored as spacer sequences, each of which is flanked by repeat sequences. Spacer repeats are transcribed into pre-crRNA (CRISPR RNAs), which are then processed into functional crRNAs^1^. Native CRISPR–Cas systems are inherently multiplexed; organisms can encode one or several CRISPR arrays and express numerous Cas (CRISPR-associated) proteins that facilitate the acquisition of new spacers and process the CRISPR arrays^2^.

Over the last few years, thousands of studies have employed CRISPR–Cas technologies to edit, or transcriptionally regulate, individual genetic loci based on their sequence complementarity with designed guide RNAs (gRNAs). Despite the general utility of CRISPR–Cas technologies, the use of an individual gRNA limits efficiencies and biotechnological applications. Therefore, there is now a growing trend in studies that move away from mono-guide approaches, and instead use multiplexed strategies for multi-locus editing or transcriptional regulation. While only four PubMed papers mentioned “multiplex” and “CRISPR” in 2013, 81 such papers were published in 2018. CRISPR systems underpinning bacterial adaptive immunity function thanks to an intricate dance between CRISPR arrays and multiple Cas enzymes; the core of this review advocates for a return to this “natural state” and emphasizes the advantages of expressing multiple Cas proteins or gRNAs simultaneously in vivo to edit or transcriptionally regulate numerous genetic loci in parallel.

In this review, we highlight strategies to create and process synthetic gRNA arrays in a multitude of organisms, emphasize the utility of inducible and orthogonal CRISPR–Cas technologies, and discuss the plethora of applications made possible by parallel targeting of genes in vivo, including biosensing, large-scale genome engineering, the construction of logic circuits, and metabolic engineering. We also argue for an enhanced focus on rigorous, quantitative experiments to fully validate and study gRNA functions in vivo, and describe the suite of computational tools available to design gRNAs. Finally, we conclude with outstanding challenges and future scientific directions ripe for innovation in the CRISPR multiplexing field.

## CRISPR for gene editing and transcriptional regulation

While many Cas proteins have been discovered, Cas9 and Cas12a (also known as Cpf1), which are RNA-guided endonucleases that cleave target DNA, are the most commonly used for genetic editing and transcriptional regulation^3^. Cas9 and Cas12a are remarkably simple to program and can be directed to DNA targets via Watson-Crick base pairing between the genetic target and gRNA. In native CRISPR systems, gRNAs for Cas9 are comprised of a crRNA:tracrRNA complex, which can be fused together via genetic engineering to form a sgRNA (single gRNA), while those for Cas12a consist solely of a crRNA^3,4^. To avoid confusion throughout this review, we use the term ‘gRNA’ to represent all CRISPR gRNA formats^4^. After forming a ribonucleoprotein complex with a gRNA, Cas9 and Cas12a perform a double-strand break adjacent to a protospacer adjacent motif (PAM), a sequence that is required for target recognition and varies between endonucleases^4^. This double-strand break enables genome editing applications.

By mutating specific amino acids in Cas9 and Cas12a, DNA cleavage activity in these enzymes is abolished, thus converting them into nuclease-null mutants, denoted as dCas9 and dCas12a^5^. Fusion of dCas enzymes to effector domains enables efficient transcriptional regulation, including CRISPR-mediated inhibition (CRISPRi) and activation (CRISPRa)^5,6^. Mechanistically, dCas enzymes repress transcription by preventing the binding of RNA polymerase or, if targeted to open reading frames, by interfering with transcription elongation^5,7^. In eukaryotes, dCas is typically fused to an effector domain to enhance repression by recruiting chromatin remodeling proteins while, in bacteria, dCas enzymes alone are usually sufficient to repress gene expression by physically blocking RNA polymerase^5,6,8^. Similarly, effector domains for CRISPRa work by recruiting RNA polymerase or by recruiting endogenous transcriptional activators^6,7,9–13^.

**Fig. 1:**
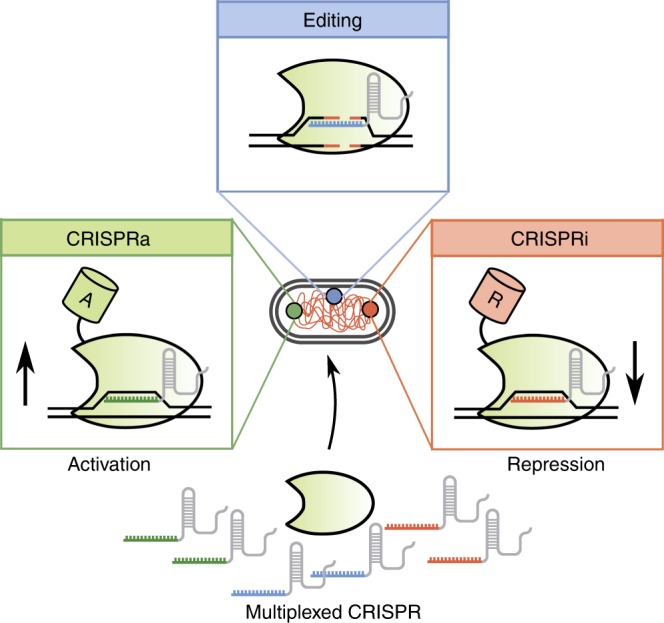
Basic overview of multiplexed CRISPR–Cas technologies. Multiplexed CRISPR–Cas can be implemented by simultaneously expressing multiple gRNAs at once. By adding orthologous Cas enzymes, gene editing, transcriptional activation (CRISPRa) and transcriptional repression (CRISPRi) can be performed in tandem at numerous locations in the genome. Cas enzymes are represented by the shaded green shape, while green and red cylinders attached to the Cas enzymes represent activation and repression domains, respectively. Editing requires that a Cas enzyme cleave dsDNA, which is repaired by error-prone, non-homologous end joining. Transcriptional regulation occurs by targeting nuclease-null Cas enzymes to specific regions up- or downstream of a transcription start site to either occlude RNA polymerase binding sites, or to recruit transcription factors (via fused effector domains) for activation or repression of the target gene.

Both CRISPR-based gene editing and transcriptional regulation benefit from multiplexing (Fig. 1). By producing multiple gRNAs and a Cas protein in vivo, researchers can build layered genetic circuits that control cellular behavior or modulate metabolic pathways with the simultaneous editing, activation, and downregulation of multiple target genes^14–17^. For gene editing, CRISPRa and CRISPRi, the targeting of multiple gRNAs to a single genetic locus also enhances the efficiency of DNA editing and transcriptional control^18–20^.

Importantly, many methods to express and process multiplexed gRNAs in vivo are based on crRNA-processing mechanisms found in native CRISPR systems. To process CRISPR arrays, some native systems rely on the nuclease itself (e.g., Cas12a and Cas13a), some rely on accessory proteins that are part of the final effector complex (e.g., Cas6/Csy4 as part of Cascade), and others rely upon a combination of tracrRNA (transactivating crRNA) and RNase III (e.g., Cas9, Cas12b, and Cas12c)^21^.

## Transcription and processing of gRNA arrays

While a simple approach to target multiple genetic loci at once in vivo is to simply transfect cells with preformed ribonucleoprotein complexes, many applications (discussed in the following sections) demand sustained functionality of CRISPR–Cas over time. Fortunately, in recent years, many strategies to genetically encode, transcribe and process large numbers of gRNAs in vivo have been developed.

**Fig. 2:**
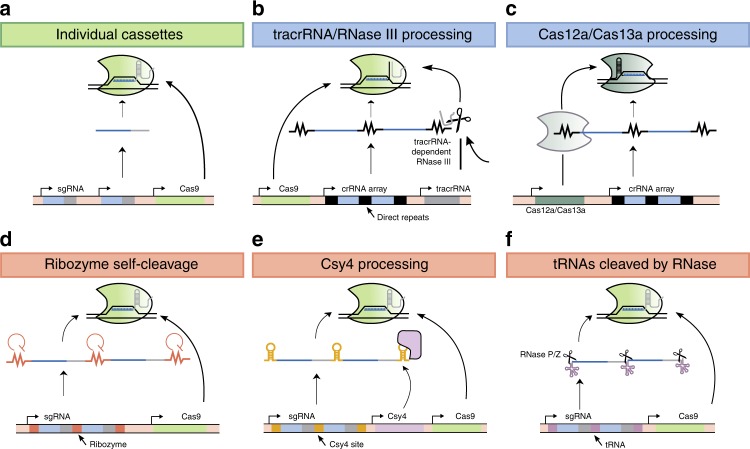
General strategies to express multiple gRNAs in vivo. The expression and processing of multiplexed gRNAs typically occur via three distinct mechanisms; arrayed sgRNA expression constructs, in which each construct contains a promoter, sgRNA, and terminator (green header), CRISPR arrays, wherein each gRNA is processed via a native CRISPR processing mechanism (blue headers), or synthetic gRNA arrays, in which each gRNA is flanked by RNA cleavage sites (red headers). **a** A common method to express numerous gRNAs in vivo is to express each gRNA from an individual promoter. **b** A strategy to process crRNA arrays, based on Type II CRISPR systems, is to flank each crRNA with a direct repeat, a repetitive sequence required for processing of pre-crRNA. tracrRNA is expressed separately. RNase III is an endogenous enzyme that removes these direct repeats in a tracrRNA-dependent manner. **c** Cas12a and Cas13a can process crRNA arrays and remove direct repeats, even in the absence of tracrRNA. **d** An engineering approach to process arrays involves flanking each gRNA with self-cleaving sequences, such as Hammerhead or HDV ribozymes. Arrays of this form have been expressed from both Pol II and Pol III promoters. **e** Csy4 is an enzyme that recognizes a 28-nt stem–loop sequence in RNA and cuts after the 20th nucleotide. Exogenous co-expression of Csy4 can be used to process gRNA arrays, provided that each gRNA is flanked by its recognition sequence. **f** gRNA arrays flanked by tRNAs can be transcribed (either by Pol II or Pol III promoters) and processed by endogenous RNase P and RNase Z, which cut the 5′ and 3′ ends, respectively, of pre-tRNAs, to produce functional gRNAs. For each sgRNA, the portion in gray is derived from the tracrRNA, while the portion in blue indicates the 20-nt spacer sequence.

There are three main “genetic architectures” for multiplexed gRNA expression. In both eukaryotes and bacteria, multiple gRNAs can be expressed by encoding each under the control of an individual promoter (typically Pol III U6 promoters in mammalian cells and Pol III tRNA promoters in yeast and plants) and terminator (Fig. 2a). Pol III promoters are high fidelity, but have a lower processivity than Pol II promoters^22^. Both Pol II and Pol III promoters have been used to express gRNAs for CRISPR–Cas studies, but Pol II promoters are easily tuned and often inducible.

In the second type of genetic architecture for multiplexed gRNA expression, gRNAs are encoded and processed by mechanisms derived from native CRISPR–Cas systems. For example, arrays of gRNAs can be processed by RNase III in a tracrRNA-dependent manner, which is the same mechanism by which crRNAs are processed in Type II CRISPR systems (Fig. 2b)^21^. This strategy enabled at least four genetic loci to be edited simultaneously in *Saccharomyces cerevisiae*, *Escherichia coli*, and cell-free extract^23^. The intrinsic crRNA-processing capabilities of Cas12a have also been leveraged to process a large number of gRNAs from a single RNA transcript, both constitutively and inducibly (Fig. 2c)^23–25^. Cas12a cleaves pre-crRNA via recognition of hairpin structures formed within the spacer repeats, producing mature crRNAs^26^. Tandem expression of Cas12a and an array of crRNAs from a single Pol II promoter in human cells enabled five target genes to be cleaved, and an additional ten target genes to be transcriptionally regulated, concurrently^24^. Multiplexed targeting via Cas12a has also been demonstrated in plants, yeast, and bacteria^27–29^. In addition, spacers for both Cas9 and Cas12a can be transcribed and processed from a single array simultaneously, with the spacers for Cas9 processed by endogenous tracrRNA-dependent RNase III^23^.

A third architecture for multiplexed gRNA expression is to produce a single transcript encoding multiple gRNAs, each separated by RNA cleavage sequences. This enhances the modularity and stoichiometry of gRNAs in vivo for CRISPR multiplexing, but demands that additional processing mechanisms are implemented to produce functional gRNAs from a single array^24^. Common strategies to accomplish this include co-expression of RNA processing enzymes, such as Csy4, or by flanking each gRNA with self-cleaving ribozymes or tRNAs.

Multiplexing can be achieved from long RNA transcripts by flanking each gRNA with the virus-derived, cis-acting Hammerhead and hepatitis delta virus ribozymes. This strategy is amenable to both Pol II and Pol III-mediated transcription and has been demonstrated in multiple organisms (Fig. 2d)^30–32^.

gRNA arrays can also be expressed from Pol II or Pol III promoters and excised by Cas family endonucleases, such as Csy4, which processes pre-crRNAs in some native CRISPR systems (Fig. 2e)^33^. Csy4 recognizes a 28-nt stem–loop sequence in RNA transcripts and cleaves after the 20th nucleotide^33^. By flanking each gRNA in an array with the Csy4 recognition sequence, multiple gRNAs can be expressed and processed from a single promoter in mammalian cells, yeast, and bacteria^30,34,35^. Our group previously expressed and processed 12 sgRNAs in *S. cerevisiae* from a single Pol II promoter with co-expression of Csy4^20^. Though Csy4-mediated processing of gRNA arrays enables a large number of gRNAs to be processed from a single RNA transcript, co-expression of Csy4 is sometimes undesirable because of cytotoxicity at high concentrations^30^.

Numerous studies have also exploited endogenous tRNA-processing machinery to process arrays of gRNAs flanked by 77-nt long pre-tRNA genes^36–38^. Processing of pre-tRNAs is mediated by ribonucleases P and Z, which cleave near the 5′ and 3′ ends, respectively, of each pre-tRNA (Fig. 2f). RNase P and Z are found in all domains of life and thus enable numerous gRNAs to be processed from tRNA–gRNA arrays in many organisms. tRNA–gRNA arrays can also be encoded in eukaryotic introns and processed by the spliceosome complex, an approach that enabled both an endonuclease (encoded in an exon) and multiple gRNAs to be expressed from a single Pol II promoter in rice protoplasts^39^.

Remarkable progress has been made in harnessing biomolecular mechanisms to produce and process increasingly long gRNA arrays. However, it is still often technically challenging to create these synthetic arrays due, in part, to the presence of highly repetitive DNA sequences.

## Methods for assembly of highly repetitive gRNA arrays

Most methods to assemble gRNA arrays do so via derivatives of popular cloning methods, such as Gibson or Golden Gate Assembly, since the repetitive sequences within gRNA arrays often make pure chemical synthesis unviable^40,41^. Assembly approaches for building synthetic gRNA arrays can be further divided into those purpose built for a specific organism, and more general strategies.

**Fig. 3:**
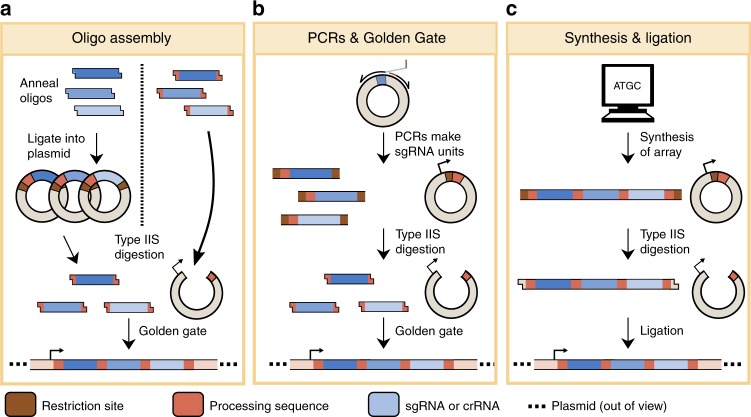
Assembly methods for synthetic gRNA arrays. **a** Oligo-based assembly methods are frequently used to assemble arrays. This approach works by annealing oligonucleotides that encode each gRNA unit, and then ligating these units into digested vectors which already contain predesigned Type IIs restriction sites and 4-nt overhangs. Each gRNA-containing entry vector is then cloned into a destination vector, which contains a promoter and terminator to express the assembled array. Conversely, oligos can be annealed that already contain complementary overhangs for direct ligation into a digested vector (shown on the right side). **b** A second strategy, referred to as PCRs and Golden Gate, utilizes a single vector with a repetitive “processing” sequence (either direct repeats, tRNA, ribozymes, or a Csy4 recognition site) as a template for PCR. Primer extensions add the desired spacer sequence and Type IIs restriction sites with specified, 4-nt overhangs. Digestion of each PCR amplicon, followed by Golden Gate assembly into a predigested destination vector, is used to assemble the final array. This strategy is both inexpensive and adaptable but requires multiple steps for cloning. **c** A more versatile, but expensive, strategy uses direct synthesis and ligation to build arrays. Briefly, multiple gRNA units are synthesized in tandem, and the ends of each synthesized piece of DNA contain overhangs for Gibson Assembly, specified Type IIs restriction sites for Golden Gate, or some other sequence to mediate DNA joining. In some cases, DNA synthesis can be directly used to directly build a full gRNA array.

Many systems to build synthetic arrays utilize plasmid-based cloning toolkits in which annealed oligonucleotides encoding gRNAs are ligated into digested plasmids with predesigned Type IIs restriction sites. Golden Gate, Gibson Assembly, or recombination is then used to join multiple gRNA-expressing entry vectors together into a digested vector, which contains a promoter and terminator for expression of the assembled array (Fig. 3a). Conversely, some methods use annealed oligos containing complementary overhangs that specify the order of each gRNA “unit”, thus enabling their direct ligation into a digested vector^23^. Array assembly methods of this type are rapid and easy to use, and have been reported for many organisms^34,38,42–45^.

Other options to produce gRNA arrays rely on a smaller set of plasmid-based parts to produce gRNAs and clone them into arrays (Fig. 3b). In these "lightweight" systems, one plasmid is typically used as a template to produce PCR amplicons, each of which is a gRNA “unit” consisting of 5′ and 3′ Type IIs restriction sites, a full gRNA, and a direct repeat, Csy4 recognition site, ribozyme, or other repetitive sequence. Units are digested and ligated into a destination vector, thus completing a functional array for in vivo gene expression^20,46^. We previously used this approach to build an array with 12 gRNAs, each flanked by Csy4 recognition sites, using two plasmids in just two days^20^. A slight variation of the same strategy also enabled the assembly of an array with 24 gRNAs, each flanked by a Csy4 recognition site^47^.

gRNA arrays flanked by direct repeats can also be constructed by ligating together synthesized DNA fragments, each containing 2–6 gRNAs (Fig. 3c). This general strategy of “synthesis and ligation” enabled the assembly of a single transcript that encodes 25 gRNAs for multiplexing in mammalian cells^23,24^. Recent efforts have also sought to reduce the number of repetitive sequences within gRNAs themselves, thus enabling the synthesis of DNA fragments encoding extra long gRNA arrays^48^. The handle of Cas9 sgRNAs (the portion that binds Cas9) is 42-nt in length and makes direct synthesis challenging. To circumvent this limitation, a recent study designed and validated a library of orthogonal, nonrepetitive Cas9 handles, promoters and terminators for *E. coli*, thus enabling up to 22 sgRNAs—each expressed from an individual promoter and terminator—to be synthesized in 3 kb chunks and ligated together^48^.

As synthetic gRNA arrays grow in length, however, it is important to consider the strategies employed to process the RNA transcripts. With long arrays, Pol III-based methods, including tRNA-mediated processing, quickly become unfeasible due to limitations on processivity (only up to eight gRNAs have been produced from this type of array)^38^. The best strategies to achieve higher multiplexing of gRNAs thus far seem to rely either on co-expression of Csy4 or on the self-processing and targeting capabilities of Cas12a^20,24^. Both of these strategies are used by native systems to produce and cleave CRISPR arrays, lending credence to their ability to efficiently process very long, repetitive RNA transcripts^3,33,49^.

## Efficiencies of multiplexed gene editing, CRISPRi, and CRISPRa

Cas enzymes can either be programmed to target many genes at once, or multiple gRNAs can be directed to a single genetic locus to enhance the efficiency of editing or transcriptional regulation (Table 1). A recent study in mammalian cells, for instance, demonstrated that the efficiency of Cas12a-mediated editing with 10 gRNAs directed to a single locus is about 60%, whereas individual gRNAs had much lower efficiencies (ranging from 2 to 17%)^24^.

**Table 1:** Assembly methods and efficiencies for multiplexed CRISPR systems.

CRISPR assembly system	Maximum sgRNAs assembled	Efficiency of edits (E) or transcriptional repression (R) or activation (A) from a single construct	Promoters used	Method of processing	Assembly method	Validated organisms	Endonucleases tested	Reference
McCarty et al.	12	(R) 3 targets, 81-95%	Single Pol II	Csy4 cleavage	Golden Gate	*S. cerevisiae*	Cas9	^20^
GTR-CRISPR	8	(E) 8 targets, 87%	Two Pol III Promoters	tRNA Processing	Golden Gate	*S.* *cerevisiae*	Cas9	^38^
CRATES	7	(E) Plasmid-clearance assays, 4 targets, >100-fold	Single Constitutive Promoter	Endogenous tracrRNA/RNase III for Cas9, Endogenous nuclease processing for Cas12a/Cas13a	crRNA *Trimming*, Golden Gate	*S. cerevisiae*, *E. coli*, cell-free	Cas9, Cas12a, Cas13a	^23^
Mans/Rossum	6	(E) 6 targets, 65%	Multiple Pol III	Individual cassettes	Gibson Assembly	*S. cerevisiae*	Cas9	^109^
Lightning GTR-CRISPR	6	(E) 4 targets with 96%, 6 targets with 60%	Two Pol III Promoters	tRNA Processing	Golden Gate	*S. cerevisiae*	Cas9	^38^
HI-CRISPR	3	(E) 3 targets, 100%	Single Pol III	Endogenous tracrRNA/RNase III	Golden Gate	*S. cerevisiae*	Cas9	^110^
CasEMBLR	5	(E) 1-5 targets, 50-100%	Multiple Pol III	Individual cassettes	Recombination in vivo	*S. cerevisiae*	Cas9	^111^
CRISPR-LEGO	4	(R) 2 genes, >90%	Single Pol II	tRNA Processing	Golden Gate	*S. cerevisiae*	Cas9	^45^
Lian et al.	3	(E) >90%, 1 gene; (R) 10-fold, 1 gene; (A) 5-fold, 1 gene	Single Pol II	Csy4 cleavage	Golden Gate	*S. cerevisiae*	Cas9, Cas12a	^15^
CAM	3	(E) 3 targets, 64%	Multiple Pol III	Individual cassettes	Recombination in vivo	*S. cerevisiae*	Cas9	^112^
CRISPRm	3	(E) 1-3 targets, 81-100%	Single Pol III	Self-cleaving ribozymes	Digestion + Ligation Cloning	*S. cerevisiae*	Cas9	^113^
Generoso et al.	2	(E) 3 targets, >90%	Multiple Pol IIII	Individual cassettes	Gibson Assembly	*S. cerevisiae*	Cas9	^114^
ELSAs	22	(R) 9 genes, 3-fold to 162-fold	Individual Promoter for each gRNA	Individual cassettes	DNA Synthesis, Gibson Assembly	*E. coli*	Cas9	^48^
CRISPathBrick	5	(R) 2 targets on 1 gene, 90-99%	Single Constitutive Promoter	Endogenous tracrRNA/RNase III	Golden Gate	*E. coli*	Cas9	^43^
Vad-Nielsen et al.	30	(R) 5 genes, 30-60%	Multiple Pol III	Individual cassettes	Golden Gate	*HEK293T*	Cas9	^44^
Campa, Weisbach et al.	25	(R) 10 genes, 35-90%, (E) 5 genes, 7-17% indels	Single Pol II	Cas12a-mediated processing	Gene Synthesis and Golden Gate	*HEK293T*	Cas12a	^24^
ASAP	10	(E) 10 genes, 50%	Multiple Pol III	Individual cassettes	Golden Gate	*HEK293T*	Cas9	^115^
Kurata et al.	10	(E) 10 targets, 0-18% indels	Single Pol III	Csy4 cleavage	Golden Gate	*HEK293T*	Cas9	^34^
Sakuma et al.	7	(E) 7 genes, 4.3-36.7%	Multiple Pol III	Individual cassettes	Golden Gate	*HEK293T*	Cas9	^116^
MuLE	4	(E) 3 genes, 50% edited at all 6 alleles	Multiple Pol III	Individual cassettes	Gateway (attL-attR Recombination)	*HEK293T*	Cas9	^117^
Kabadi et al.	4	(E) 4 genes, 17.9-33.3% in HEK293T; (E) 4 genes, 4.8-18.4% in fibroblasts	Multiple Pol III	Individual cassettes	Golden Gate	*HEK293T, Fibroblasts*	Cas9	^118^
STAgR	8	(A) 3 targets, 6-fold to 380-fold activation	Multiple Pol III	Individual cassettes	Gibson Assembly	*HeLa*	Cas9	^46^
Yin et al.	5	(E) 2 genes, 15-30% indels	Multiple Pol III	Individual cassettes	Golden Gate	Zebrafish	Cas9	^119^
Ma et al. Plants	8	(E) Multiple plasmids, 46 targets, average 85.4% mutation	Multiple Pol III	Individual cassettes	Golden Gate or Gibson	Rice, Arabidopsis	Cas9	^120^
Xie et al.	8	(E) 5 genes, 50%	Single Pol III	tRNA Processing	Golden Gate	Rice	Cas9	^36^
Xing et al.	4	(E) 2 genes, 67%	Multiple Pol III	Individual cassettes	Golden Gate or Gibson	Maize, Arabidopsis	Cas9	^42^

^#^This table is an updated, expanded version based upon a prior research article^38^. It is ordered by organism from largest to smallest number of gRNAs expressed (*E*, editing; *A*, activation; *R*, repression).

In most cases, however, it is desirable to direct gRNAs to multiple genetic loci at once. Targeting of Cas9 to ten different genes in human cells simultaneously has demonstrated that gRNAs expressed from arrays mediate genetic disruptions at efficiencies similar to gRNAs tested at each locus individually^34^. In rice protoplasts, four chromosomal deletions (eight total loci) have been performed simultaneously with 4–45% efficiency^36^. In *S. cerevisiae*, gRNA arrays have been used to implement up to eight simultaneous gene disruptions at once, with 86.7% of cells having all eight disruptions^38^. At least four genes have also been disrupted concurrently in *E. coli* with efficiencies greater than 30%^50^.

Multiplexed gRNAs are not only beneficial for gene editing, though. gRNA arrays are increasingly being utilized to tune the transcription of multiple genetic loci in tandem. In human cells, for example, ten genes have been transcriptionally repressed at once with dCas12a-based CRISPRi, with efficiencies ranging from 40 to 80%^24^. Our lab has previously expressed up to 12 sgRNAs from a single promoter in *S. cerevisiae*, targeting 4 sgRNAs to three distinct genes in parallel, with transcriptional repression efficiencies between 81 and 95%^20^. In *E. coli*, four loci have also been repressed simultaneously by dCas12a by about 50-fold each^51^.

CRISPRa applications also benefit from multiplexing. In human cells, dCas12a-based activators with three gRNAs targeting a single gene increased gene activation by 9- to 40-fold relative to single gRNAs while, with dCas9-VPR (a tripartite activator comprised of VP64–p65–Rta), four sgRNAs targeting a single gene enhanced gene activation by more than 1000-fold^11,18,19,52^. Three to sevenfold activation of three genes in rice protoplasts has also been achieved, but required a modified dCas9-fusion protein^53^.

To ensure efficient editing or transcriptional regulation by multiplexed gRNAs, predictive algorithms and computational tools to design optimal gRNAs will prove crucial. Considerable progress has been made in designing computational tools and incorporating biological factors into de novo predictions of gRNA efficiencies, but further developments are required (Box 1).

Box 1 Computational methods for designing sgRNAs For efficient editing or transcriptional regulation, well-designed gRNAs, with high efficiencies (on-target scores) and specificities (off-target scores), are required. This need is further compounded in the context of multiplexing, where multiple gRNAs edit or regulate numerous genetic loci in tandem. Target sites are easily identified by bioinformatic tools, whereby a genome is scanned to find a 18–23 bp region adjacent to a PAM site. It is much more challenging, however, to predict the on- and off-target score of each gRNA de novo. The on-target score of a gRNA is a measure of its ability to bind to its specific target site, while off-target scores predict the likelihood that a gRNA will bind other, undesired positions in the genome^121^.

On-target scores are often generated by transforming large libraries of gRNAs, together with a specific Cas protein, into cells and then reading out the indels via genome-wide profiling methods, such as Digenome-seq, GUIDE-seq and BLESS^122–124^. Genome-wide libraries of gRNAs have been developed and tested in many organisms, including rats, mice, human cells, *S. cerevisiae*, and *E. coli*^125–129^.

Off-target rules can be generated simply by scanning the genome for similar target sites or, like on-target scores, can be based on genome-wide screens. Most predictive models for off-target scores incorporate the position, number, and identity of mismatches between the spacer and DNA at non-target positions^125,130^.

Many algorithms for estimating gRNA efficiencies now extend beyond datasets comprising indel cutting efficiencies, and additionally incorporate chromatin, position, gRNA stability, Cas9 loading and sequence features of the genetic locus into the predictive model^131,132^. Biophysical models for CRISPR–Cas9 activity have also been developed, which leverage statistical mechanics and kinetics to model each step and factor in Cas9-mediated cleavage, including ribonucleoprotein formation and DNA supercoiling^133^. There are also online databases (notably WeReview: CRISPR Tools) that provide updated lists of bioinformatics tools available for CRISPR–Cas experiments^134^.

A variety of gRNA design tools are either freely available online or are integrated into DNA analysis software, such as Benchling or Geneious. Generally, these tools allow users to choose from hundreds of reference genomes, select PAM sites, target site, and receive predictions for gRNA efficiencies (based on multiple scoring algorithms), the likelihood of observing off-target effects and, in some cases, the percentage of clones that will carry frame shifts^135^. Many of these design tools have been reviewed elsewhere^136^.

The efficiency and specificity of any given gRNA is intimately dependent upon its context; expression levels, the organism in which it is expressed, the Cas enzyme, accessibility of the genetic locus and other factors all affect function. This context-dependency necessitates additional experimental datasets and computational algorithms for more precisely predicting gRNA efficiencies, especially for multiplexed applications^101,132^.

Recently, tools to optimize gRNAs based on less used endonucleases, particularly Cas12a and certain Cas9 orthologs, have also been developed, many of which use deep learning frameworks to predict activity based on large training sets^137–139^. These studies are essential because gRNAs behave differently when complexed with different endonucleases, and predictive algorithms should therefore be based on experimental data collected within a desired context.

Unfortunately, the bulk of algorithms available are still based on *editing* efficiencies, even though gRNAs for transcriptional regulation require vastly different targeting rules. Current gRNA design tools for CRISPRi and CRISPRa, such as CHOPCHOP v3, integrate positional factors into design rules, while a machine learning-based algorithm additionally incorporates nucleosome positioning, sequence features and other factors to predict highly active gRNAs^130,131^.

Recently, a computational tool to regulate many genes simultaneously in prokaryotes has been developed. This tool designs gRNAs with orthogonal Cas9 handles that can target between 1 and 20 input sequences^48^. Future computational tools made specifically for gRNA arrays should incorporate retroactive effects, gRNA; gRNA interactions, and many other factors. We are hopeful that the acquisition of additional, large-scale datasets, particularly those tested with varying Cas enzymes and in diverse organisms, will elucidate the underlying design principles of gRNAs regardless of the context in which they are expressed. The ability to predict gRNA efficiencies based solely on its expression level, the Cas enzyme used, and the context of a genetic locus—including nucleosome positioning, target position relative to transcription start site, and other factors—may be possible in the future, but will require careful measurements and well-validated datasets (Box 2).

## Orthogonal and inducible CRISPR–Cas technologies

As the number of gRNAs in a biological system increases, researchers must implement control mechanisms to limit cross talk between components and minimize off-target effects. Additional control over CRISPR–Cas can be implemented by expressing multiple Cas orthologs in vivo, by creating inducible systems wherein CRISPR–Cas components are expressed only when they are needed, or via direct engineering of gRNAs (which has been reviewed elsewhere)^54^.

Cas orthologs from different organisms each recognize a slightly different gRNA structure, enabling orthogonal assembly of ribonucleoprotein complexes. For Cas9, the nexus and hairpin of an sgRNA dictates which Cas9 ortholog they associate with (e.g., SpCas9 from *S. pyogenes* vs. SaCas9 from *S. aureus*), and swapping the nexus and hairpin regions alters which Cas ortholog is recognized^55^. Promiscuous fusion gRNAs (fgRNAs), which can associate with either Cas9 or Cas12a, have also been developed^56^. In yeast, Cas enzymes for gene editing, CRISPRa and CRISPRi have been expressed simultaneously in vivo, and functioned orthogonally simply by expressing three different gRNA architectures^15^.

Other strategies to minimize cross talk have utilized truncated gRNAs which, in some cases, are unable to mediate cleavage of target DNA, but can still recognize a genetic locus and mediate transcriptional regulation^7^. Normal length gRNAs, on the other hand, preferentially mediate cleavage of target DNA. By encoding both short and long gRNAs from a single array, and co-expressing both active and nuclease-null versions of Cas12a in human cells, simultaneous gene editing and transcriptional regulation of 15 genes was achieved in tandem^24^.

Expression of dCas9 is toxic in bacteria, and expression of additional dCas9 enzymes could further burden cells^8,57^. To overcome this in *E. coli*, scaffold RNAs (scRNAs), which are modified sgRNAs that encode both a target sequence and an RNA hairpin that recruits specific effector proteins, were used for bifunctional CRISPRi/CRISPRa with a single dCas enzyme^9^. This strategy, in which orthogonal scRNAs are used in lieu of expressing multiple different endonucleases, is also applicable in yeast and human cells^58^. T7 polymerase, a single-subunit enzyme that recognizes a short, 17 bp promoter, has also been used for orthogonal expression of sgRNAs in different yeasts, including *S. cerevisiae* and *Yarrowia lipolytica*^59^.

Cas enzymes can also be turned “on” selectively, either to limit toxicity or to enhance programmability, by linking endonuclease expression to light- or chemical-inducible promoters^9,60^. In the near future, we suspect that strategies leveraging anti-CRISPR proteins to rapidly turn “off” endonuclease functions will also be deployed^61^. Other strategies to control CRISPR–Cas activity rely on modifications to the structure of sgRNAs; conditional gRNAs are either constitutively active and turned “off” by RNA terminator switches, or are constitutively inactive and turned “on” by an RNA trigger^62^. This strategy has been demonstrated in both bacteria and human cells, enabling programmable CRISPR–Cas activity in vivo.

sgRNA behaviors can also be controlled by appending inducible, spacer-blocking hairpins to sgRNAs, which abrogates their ability to transcriptionally regulate target genes until they are turned “on” by a genetically encoded or exogenous signal^63^. Another method for inducible activation is to flank each sgRNA with microRNA-complementary binding sites^64^. In the presence of cognate microRNAs or short interfering RNAs (siRNA), functional sgRNAs are produced via Argonaute2 (Ago2)-mediated cleavage, an enzyme that cleaves mRNAs with sequences complementary to a microRNA or siRNA^65^. Recently, “switchable” Cas12a gRNAs with 5′ extensions have also been reported in bacterial cells, which are activated in response to a single-stranded RNA trigger, including full length mRNAs^66^.

With strategies to minimize cross talk between orthogonal Cas enzymes and gRNAs facilitating the implementation of multiplexed CRISPR applications, a range of biological and engineering applications have become accessible.

## Applications enhanced by multiplexed CRISPR technologies

**Fig. 4:**
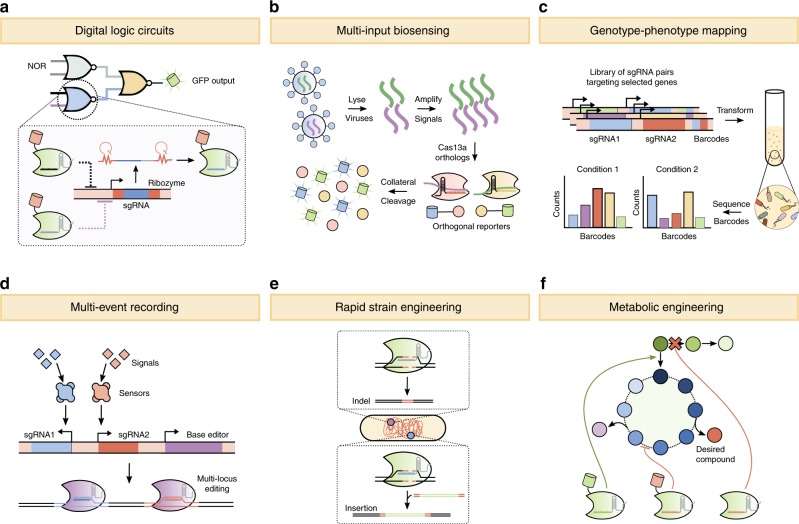
Applications of multiplexed CRISPR–Cas technologies. **a** Multiple gRNAs can be expressed, together with dCas9, to build complex logic circuits, including wired NOR gates, in which upstream gRNAs regulate the expression of downstream sgRNAs. Logic circuits can be used to produce a simple output signal, like GFP, or can be interfaced into cellular pathways to control phenotypes or behaviors. **b** Cas13a orthologs can be used to detect multiple viral pathogens at once. The viruses are lysed, their genomes are amplified, and the amplified RNA is then used as the input for Cas13a-based biosensors. Upon recognition of an RNA target, Cas13a collaterally cleaves nearby transcripts, a characteristic that can be exploited to release orthogonal, fluorescent outputs from ssRNA reporters. **c** Multiplexed gRNAs enable combinatorial mapping of genotype to phenotype. Pairs of gRNAs, each with a unique barcode, are programmed to target different genes involved in a known pathway or cellular process. These gRNA:barcode pairs are transformed into Cas9-expressing cells, and the barcodes of each cell in a population are sequenced to determine which gRNA pair each cell received. By measuring the frequency of the barcodes over multiple conditions, combinations of genes that modulate a given phenotype can be inferred. **d** Multi-event recording enables multiple signals to be detected and recorded in the genome of living cells. One gRNA is used to “write” each detected signal. Event recorders commonly use base editors and gRNAs that target a pre-defined locus, and recordings can be read out by sequencing the targeted loci. **e** Multiplexed CRISPR–Cas enables specific genomic rearrangements or modifications, including indels (which are produced by error-prone, non-homologous end joining) and insertions (via homology-directed repair, where donor DNA contains homology arms to the double-strand break), for rapid strain engineering. **f** Multiplexed CRISPR–Cas technologies can be used to perturb numerous parts of a pathway simultaneously, thus redirecting flux and enhancing the production of a desired compound. CRISPRi, CRISPRa, and editing of DNA can be achieved simultaneously, simply by expressing orthogonal dCas:gRNA pairs (one for activation and another for repression), together with Cas12a or Cas9 for editing.

Multiplexed CRISPR–Cas technologies have advanced numerous applications in basic science, synthetic biology, and biotechnology. Though there are too many applications to discuss here, we highlight those topics which specifically benefit from multiplexed strategies, such as combinatorial mapping of genotype-to-phenotype, rapid strain and metabolic engineering, multi-input biosensing, multi-event recording in living cells, and multi-layered, digital logic circuits (Fig. 4).

## CRISPR-based genetic circuits

One of the main objectives of synthetic biology is to construct genetic circuits that can control cellular behavior, but there have been serious challenges to this endeavor. A lack of well-characterized, orthogonal parts means that it is difficult to scale circuits and connect many individual regulators together^8^. The programmability of RNA:DNA base pairing, enabled by CRISPR–Cas systems, has facilitated the construction of large logic circuits with many orthogonal regulators. In yeast, 20 gRNA–promoter pairs have been shown to operate orthogonally and, in bacteria, at least 5 pairs have been demonstrated^14,16^.

Numerous studies have implemented multiplexed, CRISPR-based logic in human, yeast, and bacterial cells (Fig. 4a)^14,16,67^. For example, a central processing unit consisting of dCas9 fused to a transcriptional repressor has been used, together with multiple sgRNAs, to construct layered NOR gates in *E. coli* and numerous bitwise operators in human cells^14,67^. A NOR gate is a type of Boolean operator that produces an output signal only if both inputs are negative. A CRISPR-based NOR gate, therefore, constitutively produces an output signal (e.g., GFP or a gRNA), but is turned off when a gRNA binds to its promoter and represses transcription. In yeast, dCas9-based NOR gates with up to seven layers have been constructed^16^. In *E. coli*, NOT and NOR gates with up to five sgRNAs have also been coupled to native regulatory networks, where the output of a logic circuit is used to control a cellular phenotype, such as sugar utilization or chemotaxis^14^.

Such logic circuits have also been used to detect specific cell types and produce therapeutic payloads in response. In bladder cancer cells, for example, simultaneous activation of two cell type-specific promoters were linked to the output of different effector genes that could trigger cell death, arrest growth, or impair cell motility^68^. CRISPR-based logic circuits offer a programmable means to detect different biomarkers as inputs—which makes them a foundational tool for the assembly of more complex biosensors—and can actuate therapeutic payloads in response to such inputs^67^.

## Multi-input biosensing and diagnostics

Cas enzymes can be programmed to detect and cleave specified RNA or DNA sequences, making them a logical choice for biosensor development (Fig. 4b)^69^. Multiplexed strategies are again advantageous because the addition of gRNAs enables numerous pathogens to be detected in a single assay or multiple markers to be analyzed for a single pathogen, thus reducing costs, improving accuracy, and broadening diagnostic utility.

In vitro biosensors for viral and bacterial pathogens often rely on Cas13a (previously known as C2c2), which is a programmable RNA-guided RNase, and Cas12a^70^. Importantly, there are many orthologs of Cas12a and Cas13a^71^. Following DNA or RNA amplification^72,73^, Cas13a and Cas12a in complex with a gRNA recognize a target RNA or DNA, respectively. Upon target recognition, both enzymes nonspecifically cleave nearby RNAs or ssDNAs, respectively, in a process known as collateral cleavage^74^. By adding single-stranded RNA or DNA fused to fluorescent probes, this collateral cleavage mechanism can be exploited to conditionally produce a visible output only after formation of Cas12a/Cas13a complex hybridization with the target sequence and cleavage of the fluorescent probes, thus releasing the output signal^72,73^. By expressing multiple Cas13a and Cas12 orthologs and tuning the single-stranded linkers in these fluorescent probes, multiple reporter outputs can function orthogonally, increasing the number of pathogens that can be detected in a single assay^73^.

In vitro DNA-based biosensors have also enabled detection of pathogens, like *Mycobacterium tuberculosis*, by fusing two separate dCas9 enzymes to split halves of an output signal, such as luciferase. Each dCas9-fusion protein is programmed by sgRNAs that recognize proximal positions on an *M. tuberculosis* target sequence. Luminescence is generated when each dCas9 split-pair recognizes, and binds, to the DNA target, thus bringing the luciferase halves together^75^.

## Combinatorial mapping of genotype to phenotype

For decades, researchers have deleted genes and studied their phenotypic impact in a range of organisms, shaping our understanding of how the genome encodes information and regulates behavior. Unfortunately, rapid methods to delineate how combinations of genes contribute to a phenotype have been sorely limiting. CRISPR multiplexing can be used to probe these combinatorial, genotype-to-phenotype questions by perturbing combinations of genes in tandem, wherein sequence-based readouts of a genetic barcode (each mapped to a specific sgRNA sequence) is used to determine which cells received which sgRNAs (Fig. 4c).

To systematically dissect gene interactions responsible for ovarian cancer cell growth, one study used a library of 23,409 barcoded, gRNA pairs targeting fifty different genes encoding epigenetic regulators, based on known ovarian cancer drug targets^76^. Each gRNA was produced from an individual promoter, and lentiviral transduction of each gRNA pair, followed by serial passaging of the ovarian cancer cells and sequencing of barcode frequencies over time, elucidated pairs of gRNAs that inhibit cancer growth.

Similar combinatorial gRNA libraries have also been used with CRISPRi to explore how combinations of enhancers regulate the expression of estradiol-responsive genes and to map enhancer-gene pairs in human cells^77,78^. In the latter example, approximately 28 CRISPRi perturbations were made per cell using lentiviral transductions, and transcriptional repression of genetic loci was read out by single-cell RNA sequencing. By transducing random, triple combinations of sgRNAs, each expressed from an individual promoter, combinatorial perturbations by gRNAs were also used to determine genes involved in the unfolded protein response (a cellular stress response pathway) in human cells by screening for cells with perturbed homeostasis of the endoplasmic reticulum^79^.

## Multiplexed cellular recording

CRISPR-based bacterial adaptive immunity is a molecular recorder, whereby cells record past infections as spacers in the genome. CRISPR technologies have enabled the construction of DNA-based cellular recording devices that can provide temporal and spatial information for a milieu of biological questions (Fig. 4d)^80^.

In practice, CRISPR-based DNA memory devices require three elements: sensing of a signal, the writing of that signal into DNA, and the readout of the modified DNA (typically via sequencing of the loci) to reconstruct the information^80^. The first element, sensing, relies on protein sensors that transduce signals into the expression of a gRNA, which then serves as the ‘writing’ element to record the presence of that signal at a specified locus.

Multiplexed gRNAs can be used to record multiple input signals, including their duration, strength, and the order in which they appear^81^. CAMERA (CRISPR-mediated analog multi-event recording apparatus), for example, can simultaneously sense and record exposure to two antibiotic signals in tandem by using a base editor to convert C•G → T•A at specified loci. Further sgRNA multiplexing may enable the sensing and recording of additional signals. Other multiplexed recorders have also been developed, which can be ‘layered’ and interconnected, like genetic circuits, to both record and control molecular events in both bacterial and eukaryotic cells^82^.

## Genome, strain, and metabolic engineering

Multiplexed CRISPR–Cas technologies have been applied, with incredible success, for both rapid genome editing and for the transcriptional rewiring of metabolic pathways (Fig. 4e). CRISPR multiplexing has enabled genome editing strategies that would be difficult, or impossible, to achieve with traditional genetic engineering methods. Programmable, large-scale genome rearrangement and assembly in *E. coli* was recently performed by expressing six gRNAs; two that cut the genome and four others that cut a bacterial artificial chromosome (BAC), enabling large (greater than 100 kb) genome fragments to be rearranged at will^83^. A similar CRISPR multiplexing strategy was also used to build a chemically synthesized, 61-codon *E. coli* genome, where DNA megachunks were placed onto BACs and incorporated into the genome via multiplexed cleavage of dsDNA and lambda red recombination^84^. Genomic truncations can occur as a result of double-strand breaks, however, which may mitigate the utility of Cas9 for implementing specific mutations^85^. For precision applications requiring single nucleotide resolution, nickases and base editors, which do not induce dsDNA breaks, are preferred.

Multiplexed genome editing has also been used to create animal models for studying combinatorial genotype-to-phenotype relationships and to engineer biallelic mutations of multiple genetic loci at once^36,86^. In the largest example of multiplexing to date, 62 gRNAs were transduced into pig cells in tandem to remove all copies of porcine endogenous retroviruses, with 8% of cells having 60–100% knockout rates^87^.

In metabolic engineering, more fine-tuned approaches to gene modulation are demanded, as the flux through a pathway must be tightly regulated to enhance production of a desired chemical without impinging upon growth (Fig. 4f). Multiplexed CRISPR–Cas technologies are frequently used to simultaneously activate and repress multiple genes at once, an approach that has been used to enhance cellobiose consumption in *Yarrowia lipolytica*, isoprenoid production in *S. cerevisiae*, and n-butanol and terpenoid production in *E. coli*^88–91^. More recently, a study increased succinic acid production approximately 150-fold in *E. coli* by co-expressing an inducible dCas9 with 20 sgRNAs to knockdown 6 genes^48^. Inducible dCas9 or gRNAs enable control over the timing and magnitude of metabolic pathway regulation, and can be used to implement growth decoupling mechanisms in vivo, whereby “growth” and “bioproduction” states are toggled at will to enhance fermentation yields once a population of cells reach a desired biomass^92^. Our group has also previously used xylose-inducible, multiplexed CRISPRi with three gRNAs (targeting N-Acetylglucosamine synthesis, glycolysis and peptidoglycan synthesis) to engineer a GlcNac overproducer *Bacillus subtilis* that can simultaneously co-utilize glucose and xylose, a feat not possible by many wild-type bacteria because of carbon catabolite repression^93^.

## Insights and challenges of multiplexed crispr technologies

Despite the utility of multiplexed CRISPR technologies, serious biological and technical challenges remain. Perhaps most important, from an engineering standpoint, are the current difficulties in creating long arrays of gRNAs, and in predicting how these gRNAs will behave in living cells. The technical challenge of array assembly is rapidly being surmounted, thanks to better DNA synthesis chemistry and the development of orthogonal Cas9 handles that minimize repetitive sequences in sgRNA constructs, which were previously discussed^48^.

While we envision that current challenges in DNA synthesis and gRNA design, for example, will be overcome in the near future, other challenges will take longer to solve. One prominent problem with using CRISPR multiplexing to cut multiple genetic loci at once is the emergence of undesired chromosomal rearrangements. Fortunately, some studies have used paired sets of gRNAs to make specific cuts on chromosomes and then analyze the resulting rearrangements, and indicate that these large-scale rearrangements may be predictable in advance^94^.

Another serious challenge is that, as the number of gRNAs in a cell scales, they must compete for a dwindling ‘pool’ of endonucleases. This competition, in turn, alters the efficiency of every gRNA, an effect called retroactivity^8^. While synthetic biologists strive for modularity by combining many individually characterized genetic parts together, the interconnections between these parts often result in unpredictable outputs—this is especially pertinent for CRISPR–Cas multiplexing^95^. Retroactivity also makes it difficult to predict gRNA efficiency from sequence, another major obstacle for scaling multiplexed systems (Box 1).

Some studies have attempted to quantify the retroactive effects of multiplexed sgRNAs, and have demonstrated that titration of dCas9 with an increasing number of sgRNAs leads to predictable decreases in gRNA efficiencies^96^. In *E. coli*, sgRNAs for CRISPRi exhibited less than 10-fold repression once 7 or more sgRNAs were expressed (down from ~60-fold repression exhibited by the first sgRNA)^8^. A simple, ordinary differential equation model suggests that dCas9 bottlenecks cause CRISPRi target repression to drop off by roughly 1/*N*, where *N* is the number of sgRNAs expressed^96^. This effect was partly ameliorated in bacteria by using a dCas9-repressor fusion that recognizes a specific operator sequence and exhibits a lower toxicity, though this reduces the flexibility of DNA targeting^8^. dCas9 bottlenecking could possibly be mitigated by using conditional gRNAs, which are selectively triggered as needed in vivo^62^.

Cas9:gRNA complexes can recognize and bind genetic loci with as little as 5-nt of homology with the spacer, leading to off-target binding. Scaling the number of gRNAs within a cell enhances these off-target effects. Many sophisticated, genome-wide screens have assessed how parameters in gRNA spacer sequences, mismatches, and even the targeting of non-coding genes, can enhance off-target effects and deleteriously impact fitness for Cas9-mediated editing, CRISPRi and CRISPRa^97–99^. Fortunately, there are numerous strategies to mitigate off-target binding, including direct modification of the gRNA by shortening the spacer or truncation of one end of the tracrRNA element^100,101^. Other strategies employ mutant Cas9 endonucleases that function as nickases, with paired sets of gRNAs, which have been shown to reduce off-target effects as much as 1500-fold in certain cell lines compared to wild-type Cas9^102^. dCas9 can also be fused to FokI nuclease domains, which significantly improves target-specific editing compared to wild-type Cas9^103,104^. The recent development of search-and-replace genome editing, wherein nuclease-null Cas9 nickase is fused to an engineered reverse transcriptase, also enables DNA editing (insertions and all 12 types of base-to-base conversions) without double-strand breaks, and reduces off-target effects compared to wild-type Cas9^105^.

When working with gRNA-expressing arrays, one must account for context-dependent effects (Box 2). In some Csy4-processed arrays, for example, internally positioned gRNAs have lower expression compared to those placed on the ends of arrays^34^. gRNA arrays processed by Cas12a also vary drastically in mature RNA abundance based on their initial spatial position, likely due to the formation of stable, imperfect hairpins bridging spacer sequences^23,24^. In both cases, spatial effects can diminish gRNA expression and, in turn, efficiency.

Even in cases where numerous high-efficiency gRNAs are expressed, we still do not have a clear understanding of the limits for gRNA multiplexing in vivo. Despite our belief that hundreds of gRNAs could be expressed at once, it is unclear whether they would be effective due to increased competition for Cas enzymes. The processing efficiency of certain methods, including Csy4, ribozymes, and tRNA, will play a crucial role in determining the theoretical gRNA ceiling, and strategies to both create and process long arrays should be optimized in future studies.

Box 2 Validation strategies for sgRNA arrays: an argument for context Despite a plethora of well-validated gRNAs available in databases, the labor required to validate many gRNAs, especially when expressed from an array or for a different Cas enzyme, quickly scales when implementing multiplexed strategies.

The efficiency of each gRNA is intimately tied to its context, both spatial and temporal, and is altered depending on a multitude of factors, including retroactive effects and Cas-bottlenecking^8,99^. To construct a gRNA array that produces a desired effect, therefore, we argue that it is critical to evaluate gRNAs in their native context. Which position should each gRNA be placed in an array to obtain the desired level of expression? Which PAM site within each target gene should each gRNA be directed to? How does the expression level of each gRNA impact its overall efficiency? The answers to these questions depend intimately on the organism being studied, the Cas enzymes at play, the position of each gRNA within the array, and many other factors^132,140,141^. We argue that the best way to delineate the answers to these questions is to perform quantitative experiments.

There are numerous methods to “quantify” CRISPR-mediated editing, including a number of commercially-available mutation detection kits^142^. However, it is harder to rigorously (and consistently) quantify the efficiency of CRISPRi and CRISPRa experiments, particularly because gene expression is impacted by many intrinsic and extrinsic cellular processes. Despite this difficulty, the rigorous characterization and testing of each gRNA is absolutely crucial for the CRISPR multiplexing field moving forward, as high-quality, replicable datasets are needed to generate algorithms that can accurately predict gRNA efficiencies in a context-agnostic manner. Rigorous validation of any gRNA array used for CRISPRi and CRISPRa should therefore seek to measure two variables: the abundance of each processed gRNA in the organism, and the abundance of RNA transcripts produced from the targeted genetic loci. Both values can be determined from quantitative, next-generation sequencing. The abundance of each gRNA can be measured via small-RNA sequencing, while quantitative polymerase chain reaction or RNA-seq can be used to count the number of RNA transcripts from each target gene for both CRISPRi and CRISPRa^24^. For some applications, it may even be pertinent to quantify the abundance of functional proteins and to perform metabolic analyses, which could offer a more ‘truthful’ readout of cellular effects.

With quantitative datasets in hand, researchers will be fully equipped to mechanistically probe how the expression of any given gRNA influences its efficiency in vivo. This data will prove invaluable to enhance predictive algorithms for large-scale genomic editing and transcriptional regulation.

## Future directions

CRISPR multiplexing offers a powerful approach to answer both fundamental and applied research questions. But despite the abundance of tools and techniques available, serious challenges remain. While some organisms encode 400 or more gRNAs within their CRISPR locus, engineered strategies have thus far been limited to arrays with just 25 gRNAs. However, it is feasible that existing methods of gRNA array assembly, as outlined in this review, will enable the in vivo expression of much longer arrays. Additionally, native CRISPR systems employ many Cas enzymes in tandem, each with specified functions, to transcribe, process, and utilize these gRNAs—we envision that the same could soon be accomplished with engineered organisms.

We suspect that future developments in the CRISPR multiplexing field will be limited not by a lack of CRISPR–Cas-based tools, but rather by deficiencies in our mechanistic understanding of how things like Cas toxicity, on- and off-target effects and orthogonality between CRISPR components actually operate in vivo^106^. Ongoing advancements in basic biology and genomics, computation, and chemistry will ameliorate some of the current bottlenecks in gRNA multiplexing. Chemistries for DNA synthesis have significantly improved in recent years, and some companies are now able to synthesize long, highly repetitive sequences, which will ease current technical challenges in producing arrays^24^. In addition, cleaner datasets of gene targeting efficiency and gRNA abundance produced from standardized next-generation sequencing protocols could enable highly predictive computational models for sgRNAs embedded within arrays, accounting for processing efficiency, spatial context of the genetic loci, and other factors (Box 2). In the future, a deeper, mechanistic understanding of gRNA:DNA binding may even enable highly precise modulation of gene expression, rather than the general up- and downregulation that is common today. Multiplexed gRNAs with “non-canonical” Cas enzymes, especially Cas13a, may enable all-in-one diagnostics for specific classes of viral and bacterial pathogens^107^. As the number of gRNAs that can be expressed in vivo expands, we also envision minimal cells that operate and respond to environmental cues via entirely synthetic, programmable, transcriptional regulatory networks^67^. The ability to rewire the metabolisms and genome regulatory networks of living cells could even help shift the planet away from a petroleum-based economy. CRISPR multiplexing will also serve as a crucial tool to probe deep questions in fundamental biology, particularly as our ability to precisely control transcription improves^108^.

The pace of research in CRISPR multiplexing has been astonishing. But to continue advancing in our ability to engineer living organisms, we will require rigorous, quantitative datasets that accurately capture the milieu of factors governing the efficiency of any engineered CRISPR–Cas technology. For example, scientists could study how parameters such as gRNA abundance impinge on gene expression using quantitative RNA-seq or quantify how specific gRNAs used with CRISPRi and CRISPRa alter the abundance of proteins using mass spectrometry (Box 2). With biologists, chemists and engineers working together, and quantitative measurements in hand, we may soon have the unprecedented ability to control, rewire, and program genomes in a totally predictable manner.
